# Relationship between the drainage pattern of the dural venous sinuses and hearing recovery in patients with sudden sensorineural hearing loss

**DOI:** 10.1038/s41598-020-62946-4

**Published:** 2020-04-07

**Authors:** Jung Woo Park, Dong Hyun Kim, Tae Kyu Kang, Woongsang Sunwoo

**Affiliations:** 1 Department of Otorhinolaryngology-Head and Neck Surgery, Gachon University Gil Medical Center, Incheon, Korea; 2 Department of Otorhinolaryngology-Head and Neck Surgery, College of Medicine, Gachon University, Incheon, Korea

**Keywords:** Medical research, Outcomes research

## Abstract

Although cochlear venous insufficiency has been considered to cause sudden sensorineural hearing loss (SSHL), there is insufficient clinical evidence to support this hypothesis. We sought to determine whether there is a correlation between draining patterns of the dural venous sinuses and the side of the affected ear in SSHL, as well as hearing recovery. The medical records of 109 patients diagnosed with unilateral SSHL were retrospectively reviewed. Magnetic resonance images and pure tone audiometry were performed in all patients. We measured the dominance of the inferior petrosal sinus (IPS) and transverse-sigmoid sinus (TS/SS) ipsilateral to the affected ear. Most patients were characterized by asymmetric venous drainage (IPS, 53.2%; TS/SS, 81.7%). The dominant side of the IPS or TS/SS was independent of the side of the affected ear for all patients in this study. However, in 35 patients with early recovery within 2 weeks, the dominant side of TS/SS was significantly associated with the side of the affected ear (*p* = 0.011). Moreover, the dominance of both the IPS and TS/SS influenced hearing outcomes at 3 months. Dominant TS/SS ipsilateral to the affected ear, particularly in the presence of ipsilateral hypoplastic IPS, is associated with a favorable hearing prognosis of SSHL.

## Introduction

Sudden sensorineural hearing loss (SSHL) is frequently associated with idiopathic etiology. Several theories concerning causes of SSHL have been proposed, including viral infection and autoimmune disease. Disruption of cochlear blood flow has also been considered as one of the factors explaining the potential pathophysiology of SSHL^1^. Studies using an animal model have provided evidence to support vascular impairment as a possible cause of SSHL, since it is not possible to directly measure or visualize small amounts of blood flow in the cochlea using current techniques in humans^1^. Similar to retinal vein occlusions, impaired venous return from the cochlear circulation is suspected to cause inner ear disorders^2^. However, there are fewer experiments investigating the effect of venous congestion produced in the cochlea, compared to the number of experiments involving occlusion of arteries to the cochlea^2,3^. The inferior cochlear vein (ICV) is the main draining vein of the cochlea. The ICV runs within the canal of Cotugno proximal to the cochlear aqueduct. Considering the venous drainage of the cochlea into the dural sinus, anatomic variability of adjacent sinuses, including the inferior petrosal sinus (IPS), transverse-sigmoid sinuses (TS/SS), or the jugular bulb, may affect the venous drainage of the cochlea and the development of hearing impairment.

This theory of venous congestion is supported by the fact that the dural sinuses frequently have asymmetric drainage in the normal population. Prior studies have demonstrated that 23–35% and 72% of cases presented asymmetric structure of the IPS and TS/SS, respectively^4^. In addition, there have been radiological studies showing significant asymmetry of the jugular bulb^5^.

Contrast-enhanced magnetic resonance imaging (MRI) of the temporal bone is extremely sensitive and widely available to detect retrocochlear pathology in patients with SSHL. MRI has the additional advantage of identifying other possible causes of SSHL, such as blood vessel anomalies^6^. Recently, a relatively new modality, three-dimensional (3D) contrast-enhanced magnetization-prepared rapid gradient-echo (MP-RAGE) sequence has been reported to be useful for assessing the intracranial venous system with good contrast and resolution between the vessels and any adjacent structures^7,8^. Here, we assessed the patterns of the major venous dural sinuses, including the IPS and TS/SS, ipsilateral to the ear with SSHL using MP-RAGE sequence.

To our knowledge, no study has explored the relationship between draining patterns of the venous dural sinuses and affected ear and related outcomes in patients with SSHL. Thus, the present study sought to investigate whether there is a correlation between draining patterns of the IPS and TS/SS, as demonstrated by 3D MRI and the side of the affected ear in SSHL, as well as recovery from SSHL.

## Materials and methods

The study protocol and a waiver of consent for retrospective chart review were approved by the institutional review board of the Clinical Research Institute at Gachon University Gil Medical Center (approval no. GFIRB2019-100). All methods employed in this study were in accordance with the approved guidelines and the Declaration of Helsinki. Data were collected from an electronic medical records database and analyzed anonymously.

### Subjects

A retrospective chart review was conducted between January 2015 and December 2018 to identify patients with unilateral SSHL. SSHL was defined as a sensorineural hearing loss of greater than 30 dB occurring in at least three contiguous frequencies in less than 72 hours^9^. All patients underwent a comprehensive otoneurological examination and radiologic evaluation including temporal bone MRI, in order to rule out identifiable causes of SSHL. Patients with neurological or otological diseases were excluded from the study. Additional exclusion criteria included: (1) age less than 18 years; (2) duration of SSHL prior to treatment exceeding 7 days; (3) relapsing or fluctuating hearing loss; (4) recent head injury or acoustic trauma; and (5) preexisting asymmetric hearing loss without baseline audiometric assessment prior to SSHL. A total of 109 patients were finally included, and their MRI, audiogram, and medical records were reviewed by two otologists.

### Treatment protocols

All patients received oral corticosteroids with or without intratympanic corticosteroids. The treatment doses of oral methylprednisolone were 16 tablets, which contained 4 mg each, in a divided dose (8-4-4 tablets) for 4 days, followed by a 16 mg taper every 2 days. In 69 (63.3%) patients, intratympanic dexamethasone (5 mg/mL) injections were administered every 2 to 3 days for a total of 5 sessions as initial treatment combined with oral steroids. If audiometry confirmed complete hearing recovery, all treatments were discontinued immediately.

In 19 patients with diabetes mellitus, further work-up and individualized management of hyperglycemia was provided by the Department of Endocrinology. Blood glucose levels during steroid therapy were closely monitored and controlled with the use of oral hypoglycemic drugs or insulin according to glucose levels. In 32 patients with hypertension, vital sign monitoring, including heart rate and blood pressure, was ordered every 8 hours, and revealed an absence of serious increases in blood pressure during steroid therapy.

### Audiologic findings

The pure tone audiometry data of all patients were analyzed. Pure-tone average (PTA) was calculated using thresholds at 500, 1000, 2000 and 4000 Hz. Based on the findings of previous reports, the initial audiograms were categorized into 4 types: (1) up-sloping (the 500 Hz threshold was higher by 20 dB than the 4000 Hz one); (2) flat (less than 20 dB between the 500- and the 4000 Hz thresholds); (3) down-sloping (the 4000 Hz threshold was 20 dB higher than the 500 Hz one); and (4) profound (PTA over 90 dB)^10,11^. Pure tone audiometry was usually performed at the time of diagnosis, as well as every 2–3 days during hospitalization, at 2 weeks from the onset of SSHL, and after 3 months. Once audiometry confirmed complete hearing recovery within 2 weeks, further hearing examination was performed only at the 3-month follow-up assessment. Early response was evaluated with the last test performed within 2 weeks of the onset, and the results of assessment in the third month of treatment, or complete recovery within 2 weeks, were used to evaluate the final outcome of SSHL. Recovery of hearing was defined as a PTA less than 25 dB or within 10 dB relative to the unaffected ear, regardless of the size of gain. Patients were divided into two groups: the early recovery (ER) group (n = 35), in which recovery of hearing occurred within 2 weeks, and the non-early recovery (NER) group (n = 74).

### MRI protocol

Magnetic resonance imaging was performed at a 3.0 T scanner (Skyra; Siemens Medical System, Erlangen, Germany) with a 64-channel head coil, during the period of 1–11 days after the onset of hearing loss. The mean time between imaging and the onset of hearing loss was 4.4 ± 2.4 days, which was approximately 2 days after the initial visit. In all patients, 3D contrast-enhanced MP-RAGE sequence was performed after intravenous injection of gadobutrol (0.1 mmol/kg of body weight) in transverse and coronal orientation with the following parameters: repetition time, 1820 ms; echo time, 3.82 ms; inversion time, 900 ms; flip angle, 9°; slice thickness, 1 mm; field of view, 208 × 230; and matrix size, 256 × 209.

### MRI findings

**Figure 1:**
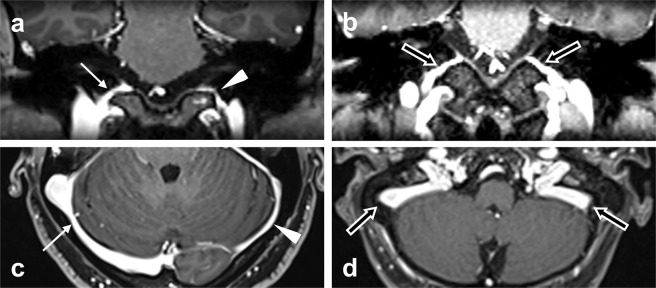
Grades of the dural venous sinuses. Coronal (**a,b**) and axial (**c,d**) 3D contrast MP-RAGE (magnetization-prepared rapid gradient-echo) images show the inferior petrosal sinuses and transverse/sigmoid sinuses, respectively. (**a,c**) If hearing loss occurs on the left side, the left sinuses (arrowheads) are not entirely visualized or are hypoplastic in relation to the contralateral side, which is classified as grade I. If hearing loss occurs on the right side, the right sinuses (white arrows) are dominant in size compared to the contralateral side, and a Grade III is given. (**b,d**) Grade II sinuses (black arrows) are approximately equal in size bilaterally.

When evaluating the draining patterns of the dural venous sinuses, a qualitative assessment was made regarding the dominance of IPS ipsilateral to the affected ear and the ipsilateral TS/SS. The dominance of sinuses ipsilateral to the affected ear was graded as follows: grade 1, aplastic or hypoplastic in relation to the contralateral side; grade 2, approximately equal in size bilaterally; and grade 3, dominant in size compared to the contralateral side (Fig. 1)^4^.

### Statistical analysis

Descriptive data were reported as count (proportion, %) or mean ± standard deviation (s.d.), as appropriate. The chi-square test, or Fisher’s exact test, was used to compare categorical variables, as appropriate for data. Continuous variables were compared using Student’s t-test. Groups for skewed variables were compared using the Mann-Whitney U test. The grades of the venous dural sinuses were compared between the ER and NER groups using the linear-by-linear association test. Statistical significance was set at *p* < 0.05. All statistical analyses were performed using SPSS software (version 19.0; IBM Corp., Armonk, NY, USA).

## Results

Our study cohort included 53 men (48.6%) and 56 women (51.4%) with an average age of 52.7 ± 12.8 years. The characteristics of 109 patients with SSHL are summarized in Table 1. Among the 109 patients with unilateral SSHL, early recovery of hearing was confirmed by pure tone audiometry in 35 patients (ER group, 32.1%). In the remaining 74 patients (NER group; 67.9%), significant hearing improvement was not observed during the 2-week follow-up period, or it did not meet the criteria for recovery.

**Table 1:** Demographic, patient characteristics.

Characteristic	Total (N = 109)	Early recovery group (n = 35)	Non-early recovery group (n = 74)	*p* value
Age (years)	52.7 ± 12.8	49.3 ± 11.7	54.2 ± 13.1	0.059^a^
Sex, male/female	53/56	21/14	32/42	0.102^b^
Side, right/left	58/51	20/15	38/36	0.572^b^
Hypertension	32 (29.4%)	10 (28.6%)	22 (29.7%)	0.901^b^
Diabetes	19 (17.4%)	5 (14.3%)	14 (18.9%)	0.552^b^
Cardiovascular disease	6 (5.5%)	0 (0%)	6 (8.1%)	0.174^c^
Vertigo	24 (22.0%)	1 (2.9%)	23 (31.3%)	**0.001**^a^
Initial PTA (dB)	72.6 ± 26.0	52.3 ± 18.2	82.3 ± 23.5	**<0.001**^a^
Final PTA (dB)	44.0 ± 31.0	14.4 ± 7.5	57.9 ± 27.9	**<0.001**^a^
Initial audiogram type				
Up-sloping	24 (22.0%)	18 (51.4%)	6 (8.1%)	**<0.001**^d^
Flat	39 (35.8%)	12 (34.3%)	27 (36.5%)	
Down-sloping	19 (17.4%)	5 (14.3%)	14 (18.9%)	
Profound	27 (24.8%)	0 (0%)	27 (36.5%)	
Period to initial visit (days)	2.3 ± 2.1	2.1 ± 1.4	2.4 ± 2.4	0.343^a^
ITS	69 (63.3%)	14 (40.0%)	55 (74.3%)	**0.001**^b^

PTA, pure-tone average of thresholds (0.5, 1, 2, and 4 kHz); ITS, intratympanic steroid injection. Continuous data are represented as mean ± standard deviation. P values were calculated using ^a^Mann-Whitney U test, ^b^Chi-square test, ^c^Fisher’s exact test, ^d^Linear-by-linear association. Bold values reflect statistical significance.

There was no significant difference between the ER group and the NER group in terms of age (*p* = 0.059), sex (*p* = 0.102), and side of the ear with SSNHL (*p* = 0.572). In addition, there was no significant difference between groups regarding comorbidities, including hypertension, diabetes, and cardiovascular disease (*p* > 0.05). In contrast, the incidence of vertigo at the onset of SSHL in the ER and NER groups differed significantly (*p* = 0.001). Only one of the 35 patients in the ER group presented vertigo. Regarding audiologic findings, the initial PTA for the whole group was 72.6 ± 26.0 dB, with a significantly worse value in the NER group (82.3 ± 23.5 dB) compared to the ER group (52.3 ± 18.2 dB) (*p* < 0.001). Clearly, the greater hearing loss and the addition of vertigo in the NER group implied that this was a more significant injury. Indeed, better outcome in the final PTA values was observed for patients in the ER group (14.4 ± 7.5 dB), with good initial hearing, than in the NER group (57.9 ± 27.9 dB) (*p* < 0.001). Regarding the initial audiograms, the flat audiogram (35.8%) was the most common type. However, the up-sloping type (51.4%), such as low-frequency hearing loss, was the most common in the ER group. As expected from the initial PTA results, the profound type (36.5%) was the predominant type in the NER group. These relationships between the initial audiogram type and hearing recovery are consistent with those reported in previous studies^10,11^.

The mean period from the onset of hearing loss to the initiation of steroid therapy was 2.3 ± 2.1 days (range, 0–7 days), with no significant differences between the 2 groups (*p* = 0.343). A comparison of combined therapy with oral steroid treatment revealed that 14 of 35 (40.0%) patients in the ER group received intratympanic steroid injection (ITS), whereas 55 of 74 (74.3%) patients in the NER group received ITS (*p* = 0.001). Further analysis was performed to confirm whether concurrent ITS therapy had a significant impact on hearing recovery. We compared 69 patients treated with concurrent ITS with 40 patients who were treated with systemic steroids alone (Table 2). Age, sex, and comorbidities did not differ statistically between the 2 groups. Although the concurrent ITS group had slightly higher thresholds (*p* = 0.014), both groups showed a similar range of values for the initial PTA. Furthermore, improvements in hearing related to the initial hearing level were not significantly different between the two groups regardless of initial PTA values (*p* = 0.057). These findings indicated that the variability of the treatments may not impact the results of the current study.

**Table 2:** Comparison of patient characteristics and hearing improvement related to the treatment protocols.

Characteristic	Patients treated with concurrent ITS (n = 69)	Patients treated with steroids alone (n = 40)	*p* value
Age (years)	51.6 ± 14.4	54.5 ± 9.4	0.203^a^
Sex, male/female	31/38	22/18	0.311^b^
Side, right/left	34/35	24/16	0.279^b^
Hypertension	18 (26.1%)	14 (35.0%)	0.325^b^
Diabetes	12 (17.4%)	7 (17.5%)	0.988^b^
Cardiovascular disease	5 (7.2%)	1 (2.5%)	0.411^c^
Vertigo	19 (27.5%)	5 (12.5%)	0.093^c^
Initial PTA (dB)	77.0 ± 27.2 (20.0–120.0)	65.1 ± 22.0 (26.5–117.5)	**0.014**^a^
**Initial audiogram type**			
Up-sloping	14 (20.3%)	10 (25.0%)	0.056^d^
Flat	22 (31.9%)	17 (42.5%)	
Down-sloping	10 (14.5%)	9 (22.5%)	
Profound	23 (33.3%)	4 (10.0%)	
Hearing improvement (dB)	26.3 ± 20.0	33.7 ± 18.0	0.057^a^

PTA, pure-tone average of thresholds (0.5, 1, 2, and 4 kHz); ITS, intratympanic steroid injection. Continuous data are represented as mean ± standard deviation. Ranges of values are given for initial PTA. Hearing improvement (dB) was calculated at the 3-month assessment relative to the initial PTA. P values were calculated using ^a^Student’s t-test, ^b^Chi-square test, ^c^Fisher’s exact test, and ^d^Linear-by-linear association. Bold value reflects statistical significance.

The anatomic variations of the dural venous sinuses are summarized in Table 3. When assessing the dominance of the IPS and TS/SS ipsilateral to the ear with SSHL, 30.3% and 41.3% were grade 1, 46.8% and 18.3% were grade 2, and 22.9% and 40.4% were grade 3, respectively. For all patients included in the present study, the dominant sides of both IPS and TS/SS showed a relatively symmetrical distribution, and no significant correlation with the side of the affected ear was found. However, a statistically significant difference between the ER and NER groups was observed for the grade of the TS/SS (*p* = 0.011). The TS/SS ipsilateral to the affected ear was dominant in 60.0% of the ER and 31.1% in the NER group. In addition, the 3-month outcome showed that there was a statistically significant relation between the dominance of the TS/SS and a high recovery rate (56.8%) (*p* = 0.026) (Table 4). In contrast, only 5 (14.3%) in 35 patients with early recovery showed dominance regarding the IPS, while 13 patients (37.1%) had hypoplastic IPS; also, significantly higher recovery rate (57.6%) was observed in the hypoplastic IPS group (*p* = 0.020) (Table 4).

**Table 3:** Draining patterns of the dural venous sinuses.

	Total (N = 109)	Early recovery group (n = 35)	Non-early recovery group (n = 74)	*p* value
**IPS grade**				
Grade 1 (hypoplastic)	33 (30.3%)	13 (37.1%)	20 (27.0%)	0.126^a^
Grade 2 (equal)	51 (46.8%)	17 (48.6%)	34 (45.9%)	
Grade 3 (dominant)	25 (22.9%)	5 (14.3%)	20 (27.0%)	
**TS/SS grade**				
Grade 1 (hypoplastic)	45 (41.3%)	10 (28.6%)	35 (47.3%)	**0.011**^a^
Grade 2 (equal)	20 (18.3%)	4 (11.4%)	16 (21.6%)	
Grade 3 (dominant)	44 (40.4%)	21 (60.0%)	23 (31.1%)	

IPS, inferior petrosal sinus; TS/SS, transverse/sigmoid sinuses. P values were calculated using Linear-by-linear association. Bold value reflects statistical significance.

**Table 4:** Recovery rates after 3 months according to the draining patterns of the dural venous sinuses.

	Grade 1 (hypoplastic)	Grade 2 (equal)	Grade 3 (dominant)	*p* value
TS/SS	15/45 (33.3%)	5/20 (25.0%)	25/44 (56.8%)	**0.026**^a^
IPS	19/33 (57.6%)	19/51 (37.3%)	7/25 (28.0%)	**0.020**^a^

IPS, inferior petrosal sinus; TS/SS, transverse/sigmoid sinuses. Values are shown as number of patients with hearing recovery/total number of patients in each category (%). P values were calculated using Linear-by-linear association. Bold values reflect statistical significance.

**Figure 2:**
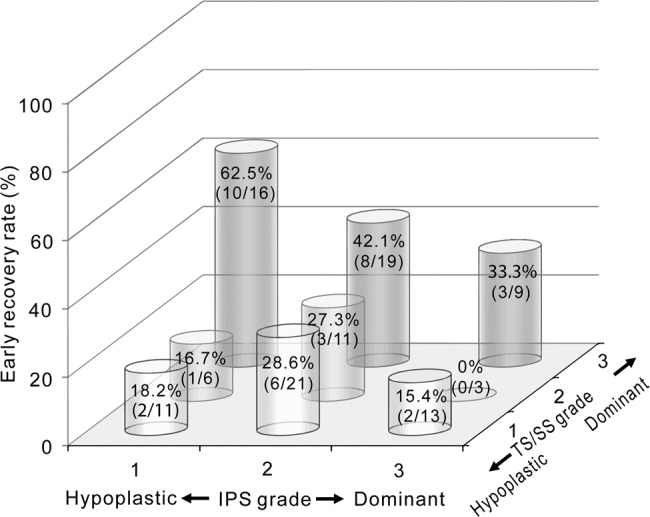
Schematic representation of early recovery rates (within 2 weeks from onset) according to the draining pattern of IPS and TS/SS. Values in each column are shown as recovery rate (number of patients with hearing recovery/total number of patients in each category).

**Figure 3:**
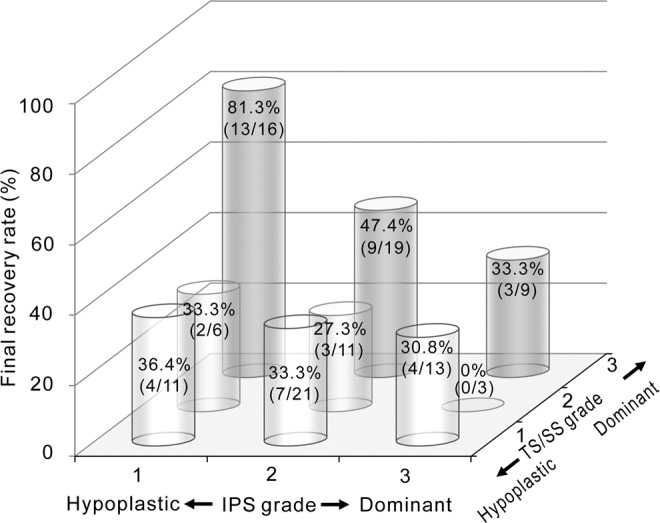
Schematic representation of final recovery rates at 3 months after treatment according to the draining pattern of IPS and TS/SS. Values in each column are shown as recovery rate (number of patients with hearing recovery/total number of patients in each category).

Therefore, we investigated whether the specific type of hypoplastic IPS with dominant TS/SS could influence the outcome and the related prognostic value by comparing of all nine possible combinations between the IPS and TS/SS for both early and final outcomes (Figs. 2 and 3). For the whole group, the mean recovery rates within 2 weeks and after 3 months were 32.1% and 41.3%, respectively. Interestingly, patients who had hypoplastic IPS and dominant TS/SS ipsilateral to the affected ear had the highest recovery rates in both early (62.5%) and final outcomes (81.3%).

## Discussion

The predominant feature of SSHL is unilateral involvement accounting for 95–98% of all patients with SSHL^12,13^. Currently, studies investigating factors that influence the determination of susceptible ear and SSHL laterality are lacking. Although the pathophysiology of SSHL remains controversial, some asymmetric anatomical conditions may be responsible for the unilateral involvement of SSHL. Since the intracranial venous system is often asymmetric and cochlear venous insufficiency has been considered to be one of the causes of SSHL, we chose to focus our study on patients with SSHL to verify whether asymmetries of the intracranial venous drainage could play a role in the etiopathogenesis of SSHL^2,3,14,15^.

On the basis of our group data, asymmetric venous drainage of the dural sinuses assessed by 3D contrast-enhanced MP-RAGE sequence proved to be very frequent (53.2% in the IPS and 81.7% in the TS/SS). The dominant side of the IPS or TS/SS was independent of the side affected by the disease for the whole group. However, we found that the dominance of ipsilateral TS/SS was significantly different between the ER and NER groups (60.0% and 31.3%, respectively). Moreover, the draining patterns of both the IPS and TS/SS influenced the outcome, which was evaluated after 3 months. Hypoplastic IPS, as well as dominant TS/SS, could be considered a favorable prognostic factor. Interestingly, the recovery rate in patients who had hypoplastic IPS and dominant TS/SS ipsilateral to the affected ear was significantly higher than in the remaining patients (62.5% versus 0–42.1% within 2 weeks and 81.3% versus 0–47.4% after 3 months).

Vascular impairment has been considered one of the widely accepted theories of the pathophysiology of idiopathic SSHL. As supportive evidence of a vascular theory, some authors reported the positive association between risk factors for ischemic vascular disease and SSHL^16,17^. However, the effect of these risk factors on the prognosis of SSHL is still controversial. Some authors have reported a negative effect of comorbid diabetes, hypertension, or hypercholesterolemia on hearing improvement in SSHL^18,19^, while other authors have reported no effect of these conditions on SSHL^20,21^; meanwhile, Orita *et al*.^22^ reported better hearing prognosis in patients with hyperglycemia. The discrepancy between the results of previous reports suggests that SSHL resulting from vascular impairment can be a heterogeneous disease, which may be caused by difference in the pathogenesis of the disease. Indeed, an impairment of cochlear blood flow can be induced by a disturbance of venous drainage from the cochlea, as well as by a disruption of arterial blood supply to the cochlea.

The cochlea requires high-energy metabolism and is supplied by only one end artery, namely, the labyrinthine artery. This type of vascularization makes the cochlea especially vulnerable to occlusion of the arteries from which the labyrinthine artery arises. It has been reported that when cochlear blood supply is interrupted by arterial occlusion, fibrosis and ossification of the cochlea typically occurs in both human and animal models^23–25^. As can be expected based on the pathological changes occurring in the cochlea, hearing loss due to total arterial occlusion is permanent and is associated incomplete recovery^26,27^. Perlman *et al*.^28^ investigated the effect of temporary total occlusion of the labyrinthine artery in guinea pigs. They reported that cochlear function as reflected in microphonic and action potentials was significantly decreased within one minute of occlusion and not recovered completely after 30 minutes of occlusion. If arterial blood supply was interrupted for over 60 minutes, there was no recovery of cochlear function, and histological changes were noted throughout the cochlea. In contrast, 45–65% of SSHL patients without identifiable cause showed spontaneous recovery of hearing within 2 weeks^29,30^. This rate of spontaneous recovery of hearing could indicate the presence of another cause of SSHL, such as venous insufficiency.

Reduced cochlear blood flow due to venous insufficiency is another theoretical mechanism by which vascular impairment can induce cochlear dysfunction. Cerebral venous thrombosis, chronic cerebrospinal insufficiency, and hyperviscosity occurring in macroglobulinemia are known causes of reversible SSHL^12,31,32^. In addition, Gatehouse *et al*.^33^ reported a significant relationship between hyperviscosity and sensorineural hearing loss. Hyperviscosity, or disturbance of venous drainage, may lead to a decrease in cochlear blood flow, and low flow rate may contribute to venous thromboembolism. Watanabe *et al*. investigated the effects of acute venous congestion to the cochlea in the guinea pig^2,3^. They occluded the ICV, which collects all the tributaries of the entire cochlea, and observed changes in cochlear blood flow. After occlusion of the ICV, the cochlear blood flow was reduced to 60% of the baseline value, but no remarkable changes were observed histologically in the cochlea. The endolymphatic and perilymphatic pressure increased simultaneously but recovered after 5 minutes. They also found that the ICV had communicating veins from the mucoperiosteum of the middle ear and suggested that these may act as collateral veins in acute cochlear venous congestion. Due to the presence of collateral venous drainage of the cochlea, it can be assumed that the cochlea is less vulnerable to venous occlusion than arterial occlusion. Therefore, we hypothesized that cochlear venous congestion may play an important role in cases of idiopathic SSHL presenting early recovery. Our findings in the early recovery group were consistent with those of previous studies; the complaints of vertigo and hearing thresholds on initial audiogram were fewer and lower than those in the non-early recovery group^29^.

The ICV usually drains into either the IPS or directly into the jugular bulb. Subsequently, the IPS empties into the jugular bulb with various communicating branches. Since the jugular bulb is the connecting link between the sigmoid sinus and the internal jugular vein, the dominance of these ipsilateral venous structures is concordant. However, it has been reported that there was no relationship between the IPS and ipsilateral TS/SS in previous anatomical studies in the general population^4^. Similarly, only 28.4% of our patients were found to have a correspondence between the grade of the IPS and the ipsilateral TS/SS. From an anatomical point of view, the drainage configuration of hypoplastic IPS might affect cochlear venous drainage, which seems to be consistent with our hypothesis of venous vascular etiology. If the pressure gradients along the IPS are held constant and are similar on both the ipsilateral and contralateral sides, venous flow through the IPS can be different considering the variables in the Bernoulli–Poiseuille equation. Theoretically, a small cross-sectional area in hypoplastic IPS implies an increase in viscous resistance to flow^34^. Therefore, hypoplastic IPS might be associated with cochlear venous insufficiency in a subset of patients with SSHL. However, since the IPS is generally associated with marked anatomic variation, regarding both size and anastomoses, the incidence of hypoplastic IPS in the ER group could not significantly differ from that in the NER group. Surprisingly, the drainage configuration of dominant TS/SS appeared to be associated with early recovery of hearing. Dominant TS/SS is commonly accompanied by a large jugular bulb and associated enlarged jugular fossae. The size and position of the jugular bulb are variable. As an extreme example of a normal variant, a substantially enlarged jugular fossa has been reported and is related to sensorineural hearing loss^35^. In this case, magnetic resonance angiography showed turbulent flow within the jugular bulb. The pressure of the large jugular bulb to surrounding structures may also cause various symptoms. Wadin^36^ revealed that the cochlear aqueduct is the structure most frequently affected by a high jugular fossa. Based on the findings of these studies, we theorize that the turbulent flow and pressure effects within the large jugular bulb compromise the venous drainage of the cochlea by venous stasis or reflux in the IPS. As expected from venous etiology, this study revealed that patients with dominant TS/SS in combination with hypoplastic IPS had a high recovery rate of 81.3%. Thus, we suggest the possibility that dominant TS/SS, as well as hypoplasia of the IPS, may also be a potential predisposing factor for developing a substantial amount of venous blood stagnation in the compartment. Chronic venous stasis affects the function of the venous endothelium and further reduction of venous flow related to thrombosis formation, inflammation, infection, or immunological factors could lead to sudden cochlear dysfunction^37^. However, further studies are needed to investigate the association between such asymmetric venous configurations and reflux in the IPS. A reversal of flow direction during normal breathing on transcranial color-coded duplex sonography can confirm reflux in the IPS^38^.

There were some limitations to our study. First, we were only able to qualitatively assess the dominance of the venous sinuses ipsilateral to the affected ear. In this study, we used a 3D contrast-enhanced MP-RAGE sequence to evaluate the patterns of the IPS and TS/SS. Although this sequence is known to be good in the depiction of normal cerebral venous anatomy, it is very difficult to accurately measure the diameter of the venous sinuses at a consistent point due to variations with regards to the courses and branching patterns of these anatomic structures. Second, this study was limited by its retrospective design. It was not possible to reduce selection bias regarding the treatment strategy, although there is insufficient evidence to support the benefits of simultaneous intratympanic dexamethasone injection over systemic steroid therapy alone. Despite this study design, the selection bias may have been minimized, since patients were allocated randomly to one of four physicians, and every physician consistently used only one protocol. Fortunately, a higher proportion of patients in the NER group underwent additional treatment in this study, which reduced the potential bias in the results. Finally, this study design yields only correlational, instead of causal, interpretations. However, the present study shows a promising approach that can be used in a prospective, large-scale study in future investigations of SSHL with venous etiology.

In conclusion, our results may support the hypothesis that acute venous congestion and/or reduced cochlear blood flow due to venous obstruction could lead to SSHL. The dominant TS/SS ipsilateral to the affected ear, especially combined with ipsilateral hypoplastic IPS, correlates with favorable hearing prognosis of SSHL regardless of the modality of treatment.
